# Comprehensive analysis of COLGALT1 in tumor microenvironment regulation and prognosis of clear cell renal cell carcinoma

**DOI:** 10.1007/s10238-026-02041-6

**Published:** 2026-02-02

**Authors:** Yicheng Guo, Bin Wang, Guixin Ding, Yanwei Zhang, Yini Wang, Xiaohong Ma, Jitao Wu

**Affiliations:** 1 Department of Urology, Yantai Yuhuangding Hospital, Qingdao University, NO. 20 East Yuhuangding Road, Yantai, 264000 Shandong China; 2 Shandong Second Medical University, Weifang, China; 3 The Second Clinical Medical College, Binzhou Medical University, Yantai, China

**Keywords:** Clear cell renal cell carcinoma, Collagen galactosyltransferase 1 (COLGALT1), Prognosis, Tumor immune microenvironment, Tumor-infiltrating immune cells, Bioinformatics analysis

## Abstract

Collagen galactosyltransferase 1 (COLGALT1), a key enzyme involved in collagen post-translational modification, has been implicated in extracellular matrix remodeling across multiple cancer types, yet its prognostic significance and relationship with the tumor immune microenvironment in clear cell renal cell carcinoma (ccRCC) remain unclear. In this study, we analyzed multi-omics datasets from public repositories to assess COLGALT1 expression patterns, clinical relevance, and prognostic value in ccRCC. Quantitative real-time PCR was performed to validate its expression in renal cancer cell lines and normal renal tubular epithelial cells. Immune infiltration profiles were characterized using multiple computational algorithms, and a competing endogenous RNA network was constructed to explore regulatory mechanisms. Our results demonstrated that COLGALT1 expression was significantly upregulated in ccRCC at both mRNA and protein levels and was positively associated with the infiltration of monocytes, T helper 2 cells, macrophages, regulatory T cells, and natural killer cells. Notably, COLGALT1 expression correlated strongly with markers of M2 macrophages, suggesting a role in promoting an immunosuppressive tumor microenvironment. These findings identify COLGALT1 as a novel prognostic biomarker and potential therapeutic target in ccRCC, highlighting its contribution to extracellular matrix remodeling and immune regulation.

**Supplementary Information:**

The online version contains supplementary material available at 10.1007/s10238-026-02041-6.

## Introduction

Clear cell renal cell carcinoma (ccRCC), representing over 70% of kidney malignancies, is characterized by metabolic reprogramming and aberrant angiogenesis [1, 2], contributing to its resistance to conventional therapies [3, 4]. Although immune checkpoint inhibitors have shown efficacy in subsets of patients [5–7], persistent limitations in predicting treatment response underscore the need for biomarkers reflecting both tumor-intrinsic and microenvironmental features.

Collagen galactosyltransferase 1 (COLGALT1), also known as Galactosyltransferase 25 domain containing 1 (GLT25D1) [8], a critical enzyme regulating collagen glycosylation [9], has been implicated in extracellular matrix (ECM) stiffening and immunosuppressive tumor microenvironment (TME) formation across malignancies such as ovarian and breast cancers [10–12]. However, despite these observations in other tumor types, the expression profile, clinical significance, and immunological role of COLGALT1 in ccRCC remain largely unexplored. To our knowledge, no study has systematically characterized its potential impact on tumor immunity, prognosis, and therapeutic response in ccRCC.

In this study, we systematically investigated the dysregulated expression of COLGALT1 across various human cancers, with a particular focus on its prognostic significance and clinicopathological correlations in ccRCC, utilizing datasets from TCGA, GEO, UALCAN, and in vitro experiments. We further explored the relationship between COLGALT1 expression and tumor-infiltrating immune cells, as well as immune-related marker genes, by integrating data from TIMER and TISIDB. Additionally, advanced tools such as ESTIMATE, CIBERSORT, and ssGSEA were applied to examine the complex interplay between COLGALT1 expression and the immune microenvironment in ccRCC. Furthermore, potential COLGALT1-interacting proteins were identified via STRING database, and functional enrichment analysis was performed on co-expressed genes. Using the competing endogenous RNA (ceRNA) framework [13–17], we also investigated lncRNA–miRNA–mRNA regulatory networks that may modulate COLGALT1 expression.

By integrating multi-omics bioinformatics with experimental validation, our study goes beyond previous pan-cancer reports by providing the first comprehensive evaluation of COLGALT1 in ccRCC. This work highlights COLGALT1 not only as a prognostic biomarker but also as a potential immunomodulatory factor that influences tumor–immune interactions and therapeutic sensitivity. These findings offer novel insights into the tumor biology of ccRCC and establish a foundation for future mechanistic and translational research.

## Materials and methods

### Data acquisition and preprocessing

Transcriptomic profiles (TPM) and clinical annotations for 537 ccRCC cases and 72 adjacent normal tissues were retrieved from the TCGA-KIRC cohort via the UCSC Xena browser (https://xena.ucsc.edu/). Data harmonization and gene symbol conversion were performed using R packages.

### Multi-platform validation

GEO database (https://www.ncbi.nlm.nih.gov/geo/) and UALCAN portal (http://ualcan.path.uab.edu/) were leveraged to validate COLGALT1 expression patterns. Protein localization patterns in normal renal versus ccRCC tissues were visualized through immunohistochemistry images from the Human Protein Atlas (HPA; https://www.proteinatlas.org/) [13]. Normal kidney tissue was used as a positive control for COLGALT1 staining, as reported in the Human Protein Atlas. For negative controls, sections were processed in parallel with the omission of the primary antibody.

### Tissue processing

16 paired ccRCC specimens and matched normal tissues were obtained intraoperatively, immediately snap-frozen in liquid nitrogen, and stored at -80 °C for downstream transcriptomic analyses.

### Transcript quantification

Total RNA isolation was performed using TRIzol reagent (Pufei, Shanghai, China), followed by cDNA synthesis with the Promega M-MLV kit (Accurate Biology, China). COLGALT1 mRNA levels were measured via qRT-PCR system (Thermo Fisher, USA) with β-actin as endogenous control. Primer sequences are listed in Supplementary Table 1.

### Clinical-pathological correlation

Associations between COLGALT1 expression and clinicopathological characteristics were visualized through boxplots generated with ggplot2, employing Kruskal-Wallis tests for statistical significance. A comprehensive summary of clinicopathological characteristics is provided in Supplementary Table 2.

### Analysis of clinical prognostic factors

The “ggpubr” and “limma” packages were used to assess and visualize the correlation between clinical prognostic factors and COLGALT1 expression. We assess overall survival (OS), progression-free survival (PFS) and disease-free survival (DFS) associated with COLGALT1, while also computing hazard ratios (HR) and p-values. Time-dependent receiver operating characteristic (ROC) curves, along with nomograms and calibration curves, were generated using the “timeROC,” “rms,” “regplot,” “survminer,” and “survival” packages to assess the predictive accuracy of 1-/3-/5-year survival outcomes. Detailed information of TCGA-KIRC cohort is summarized in Supplementary Table 3.

### Pathway enrichment

STRING [14] (http://www.string-db.org) constructed protein interaction networks (minimum confidence 0.7), while clusterProfiler performed KEGG/GO enrichment on top 20 co-expressed genes. GO analysis consists of three components: biological process (BP), cellular component (CC), and molecular function (MF).

### Immune landscape profiling

Three computational deconvolution methods—ESTIMATE [15], CIBERSORT [16], and ssGSEA [17]—were applied to quantify immune cell infiltration levels in TCGA-curated ccRCC transcriptomic profiles. To delineate the interplay between COLGALT1 expression and immune checkpoint activity, we integrated multi-platform analyses from GEPIA2 [18] (immune checkpoint co-expression) and TIMER2.0 [19, 20] (immune cell correlation mapping).

### Spatial mapping of COLGALT1 in the tumor microenvironment

To resolve COLGALT1’s cellular localization within the ccRCC ecosystem, we performed single-cell resolution analysis through the Tumor Immune Single-cell Hub 2 platform (TISCH2; http://tisch.comp-genomics.org) [21]. This resource enabled systematic interrogation of COLGALT1’s transcriptomic landscape across malignant, stromal, and immune cell subsets, leveraging its curated single-cell RNA-seq atlases and interactive visualization modules.

### Tumor-immune system interaction database

The TISIDB [22] portal (http://cis.hku.hk/TISIDB/) was leveraged to systematically profile the interplay between COLGALT1 expression and immune regulatory networks in ccRCC. This platform aggregates multi-omic datasets across 28 cancer types, enabling multi-dimensional interrogation of tumor-immune interactions.

### Construction of the mRNA–miRNA–lncRNA co-expression network

Interaction data for mRNA–miRNA and miRNA–lncRNA pairs were retrieved from the StarBase database (http://starbase.sysu.edu.cn/) [23]. For mRNA–miRNA interactions, a program number ≥ 1 was used as the inclusion criterion. Correlation coefficients, survival analyses, and differential expression levels were evaluated using R packages. Expression relationships were categorized as negatively correlated when *r* < − 0.1 and positively correlated when *r* > 0.1. Statistical significance thresholds were set at *P* < 0.01 for differential expression and *P* < 0.05 for survival analysis.

We employed R to visualize the data and construct a key lncRNA–miRNA–mRNA competing endogenous ceRNA network relevant to ccRCC. The potential ceRNA regulatory axis involving COLGALT1 was graphically represented using BioRender.

### Drug sensitivity prediction

Drug sensitivity profiling was performed by computing the half-maximal inhibitory concentration (IC50) values through the ‘pRRophetic’ computational framework [24], which estimates the drug concentration required to inhibit 50% of tumor cell viability. This predictive modeling integrated ridge regression models with batch effect correction (via the ‘sva’ package), feature selection (‘genefilter’), and normalization pipelines (‘preprocessCore’) to systematically evaluate COLGALT1-associated chemotherapeutic responses.

### Statistical framework

**Fig. 1:**
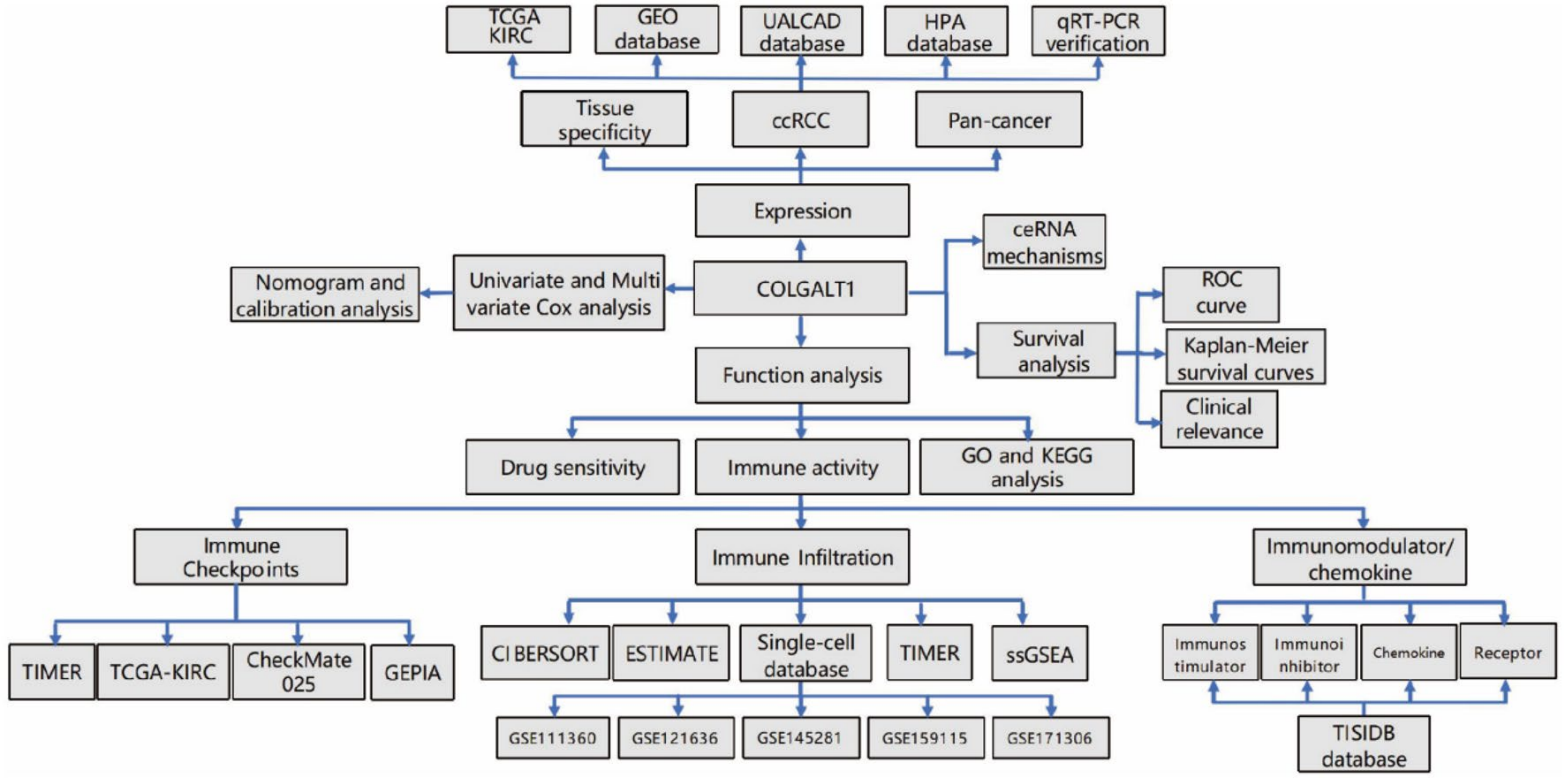
Flowchart

All statistical computations were executed using dedicated analytical suites (GraphPad Prism v9.3.1 and SPSS v26.0). Genes with an absolute log2 fold change (|log2FC|) > 1 and p-value < 0.05 (Benjamini–Hochberg correction) were considered significantly differentially expressed. These thresholds are commonly applied in bioinformatics studies to balance biological relevance and statistical rigor. All analyses were conducted in R software (version 4.4.0). Statistical thresholds were defined as *P < 0.05, **P < 0.01, and ***P < 0.001. The experimental design workflow is schematized in Fig. 1.

## Results

### COLGALT1 dysregulation in CcRCC

**Fig. 2:**
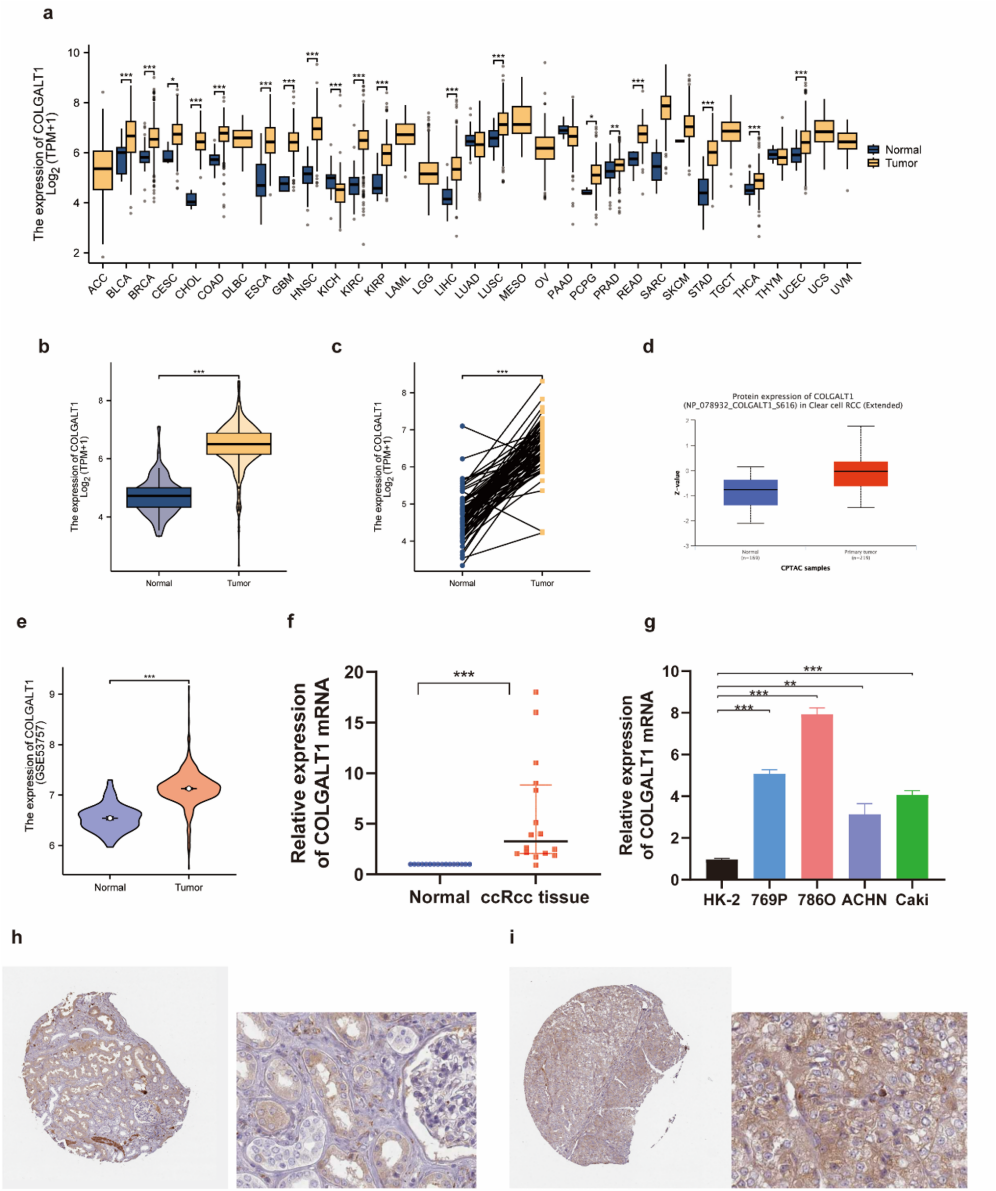
The expression of COLGALT1 in ccRCC and pan-cancer (**a**) Differential expression of COLGALT1 across various tumor types via TCGA. (**b**) mRNA levels of COLGALT1 are significantly elevated in 539 ccRCC samples compared to 72 normal tissue samples in the TCGA dataset. (**c**) COLGALT1 expression is markedly higher in ccRCC tissues than in paired normal tissues according to TCGA data (*n* = 72). (**d**) Protein-level expression of COLGALT1 based on analysis from the CPTAC database. (**e**) Analysis of matched tumor and adjacent normal samples from the GEO database revealed that COLGALT1 mRNA levels were elevated in tumor tissues. (**f**) qRT-PCR analysis was carried out on paired tumor and adjacent normal tissue samples from 16 confirmed ccRCC patients to evaluate COLGALT1 expression levels. (**g**) experiments were conducted in renal cell carcinoma cell lines (**h**) COLGALT1 protein expression in normal tissues, as presented by the Human Protein Atlas. (**i**) COLGALT1 protein expression in tumor tissues, according to data from the Human Protein Atlas. (*P < 0.05, **P < 0.01, ***P < 0.001)

Cross-cancer evaluation of COLGALT1 transcript levels was performed through TCGA datasets (Fig. 2A). This pan-cancer screening identified significant COLGALT1 upregulation in multiple malignancies, particularly cholangiocarcinoma, clear cell renal carcinoma, breast cancer, and colon adenocarcinoma compared to normal counterparts.

Focusing on ccRCC, integrated analysis of TCGA-KIRC, UALCAN, and GEO cohorts demonstrated marked COLGALT1 overexpression in tumor tissues versus normal controls (*P* < 0.001, Fig. 2B-E). Additionally, qRT-PCR analysis revealed that COLGALT1 expression was upregulated in 16 paired ccRCC tissues compared to adjacent normal tissues, and consistently elevated in multiple ccRCC cell lines (769-P, 786-O, ACHN, and Caki) relative to the normal renal epithelial cell line HK-2. (Fig. 2F, and G). HPA immunohistochemical data revealed pronounced COLGALT1 protein accumulation within malignant renal epithelia (Fig. 2H-I), aligning with transcriptomic findings. Multi-source evidence convergence confirmed COLGALT1 hyperexpression as a hallmark of ccRCC pathogenesis.

### Correlation between COLGALT1 expression and clinicopathological parameters

**Fig. 3:**
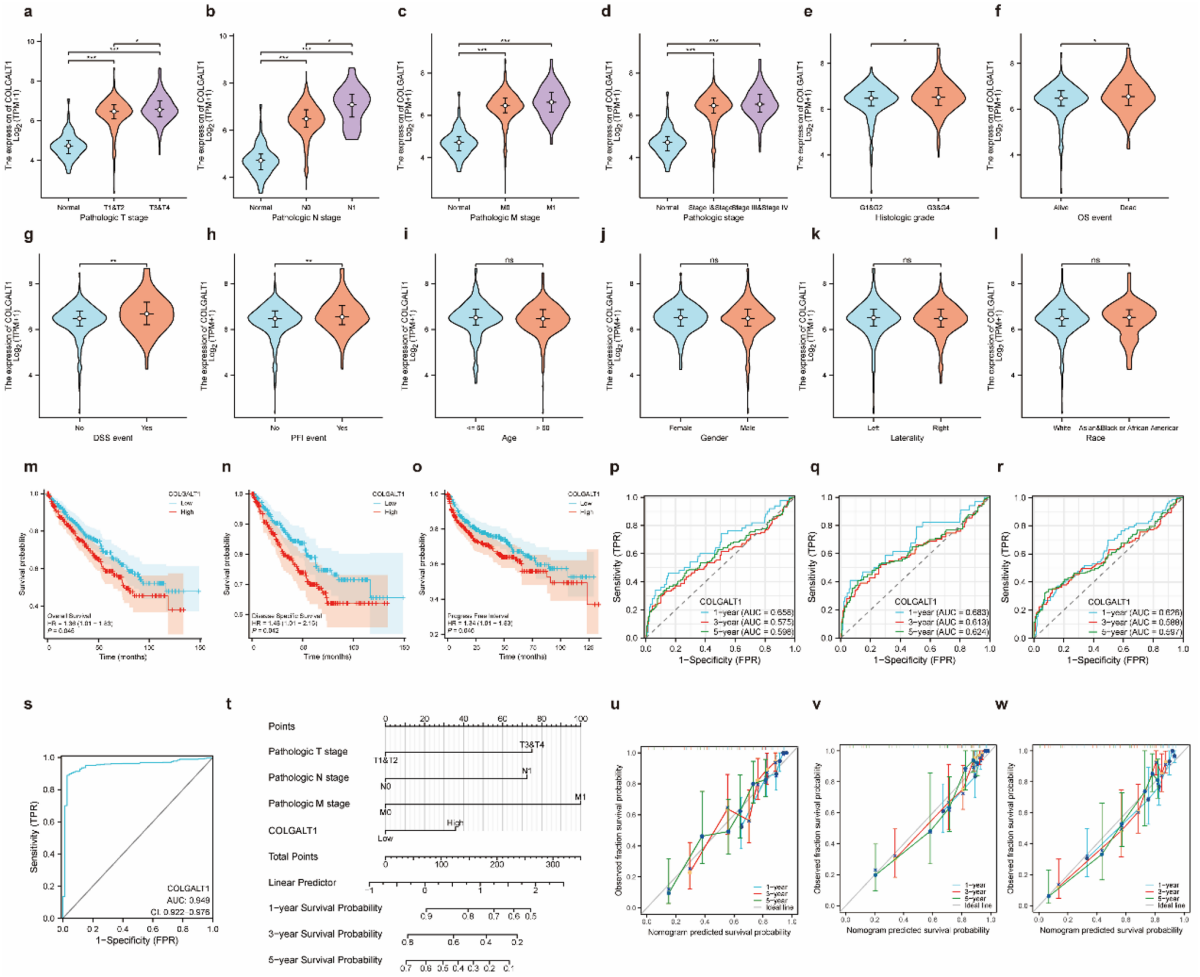
COLGALT1 expression is associated with diverse clinicopathological features in ccRCC patient **a–h** Associations between COLGALT1 expression and various clinical parameters, including: (**a**) T stage, (**b**) N stage, (**c**) M stage, (**d**) histological grade, and (**e**) pathological stage. Additional analyses demonstrate the relationship between COLGALT1 levels and (**f**) overall survival events, (**g**) disease-specific survival events, (**h**) progression-free interval events, (**i**) patient age, (**j**) gender, (**k**) tumor laterality, and (**l**) race in ccRCC cases. The Kaplan-Meier curves indicate significant differences in (**m**) overall survival, (**n**) disease-specific survival, and (**o**) progression-free interval between patients with high versus low COLGALT1 expression. Time-dependent ROC analysis further confirmed the predictive performance of COLGALT1 for (**p**) OS, (**q**) DSS, and (**r**) PFI, respectively. (**s**) displays an ROC curve in which COLGALT1 achieved an AUC of 0.949, effectively distinguishing ccRCC tissues from normal kidney samples. (**t**) presents a nomogram designed to estimate the 1-, 3-, and 5-year OS probabilities for individuals with ccRCC. Calibration plots shown in panels (**u–w**) validate the predictive accuracy of the nomogram across these three timepoints. (*P < 0.05, **P < 0.01, ***P < 0.001; ns: not significant)

Demographic and clinicopathological characteristics of the TCGA-ccRCC cohort are detailed in Supplemental Table 4. Elevated COLGALT1 expression demonstrated significant correlations with advanced T stage (*P* < 0.001), N stage(*P* < 0.001), M stage (*P* < 0.001), high histologic grade (*P* < 0.05), and aggressive pathologic stage (*P* < 0.001) (Fig. 3A-E). Notably, COLGALT1 overexpression strongly predicted unfavorable OS (*P* < 0.05), DSS (*P* < 0.001), and PFI (*P* < 0.001) (Fig. 3F-H). No significant associations were observed with age, gender, race or laterality (Figure. 3I-L).

### Value of COLGALT1 expression in diagnosis and predicting prognosis

To investigate the prognostic and diagnostic utility of COLGALT1 in ccRCC, we performed comprehensive survival analyses and predictive modeling. Survival outcomes were assessed using Kaplan-Meier methodology to evaluate the association between COLGALT1 transcript levels and clinical endpoints including OS, DSS, and PFI. As shown in Figs. 3M-O, elevated COLGALT1 expression demonstrated significant correlations with reduced survival outcomes across all measured parameters. Specifically, patients with higher expression exhibited poorer OS (HR = 1.36, 95% CI 1.01–1.83; *P* = 0.045), DSS (HR = 1.48, 1.01–2.16; *P* = 0.042), and PFI (HR = 1.34, 1.01–1.83; *P* = 0.046). Time-dependent ROC analysis revealed moderate predictive capacity for COLGALT1 in long-term outcome prediction. The AUC values for OS prediction at 1-, 3-, and 5-year intervals were 0.658, 0.575, and 0.598 respectively (Fig. 3P). Similar prognostic performance was observed for DSS and PFI assessments (Fig. 3Q, and R). Notably, COLGALT1 exhibited exceptional diagnostic discrimination between malignant and normal tissue specimens (AUC = 0.949, Fig. 3S). Multivariate regression modeling identified COLGALT1 expression (HR = 1.898, 1.396–2.519; *P* = 0.001) as an independent prognostic determinant along with established clinical parameters: advanced pathologic stage (HR = 2.706, 1.980–3.442; *P*<0.001), and higher histologic grade (HR = 1.743, 1.224–2.509; *P* = 0.002) (Supplemental Table 5). These variables were incorporated into a composite prognostic nomogram (Fig. 3T) enabling quantitative prediction of 1-, 3-, and 5-year survival probabilities. The model’s predictive accuracy was validated through calibration analysis, where predicted outcomes showed strong concordance with actual observations across all timepoints (Fig. 3U-W).

These collective findings position COLGALT1 as a dual-purpose biomarker with potential applications in both diagnostic evaluation and prognostic stratification for ccRCC patients.

### Enrichment analysis of COLGALT1 in CcRCC

**Fig. 4:**
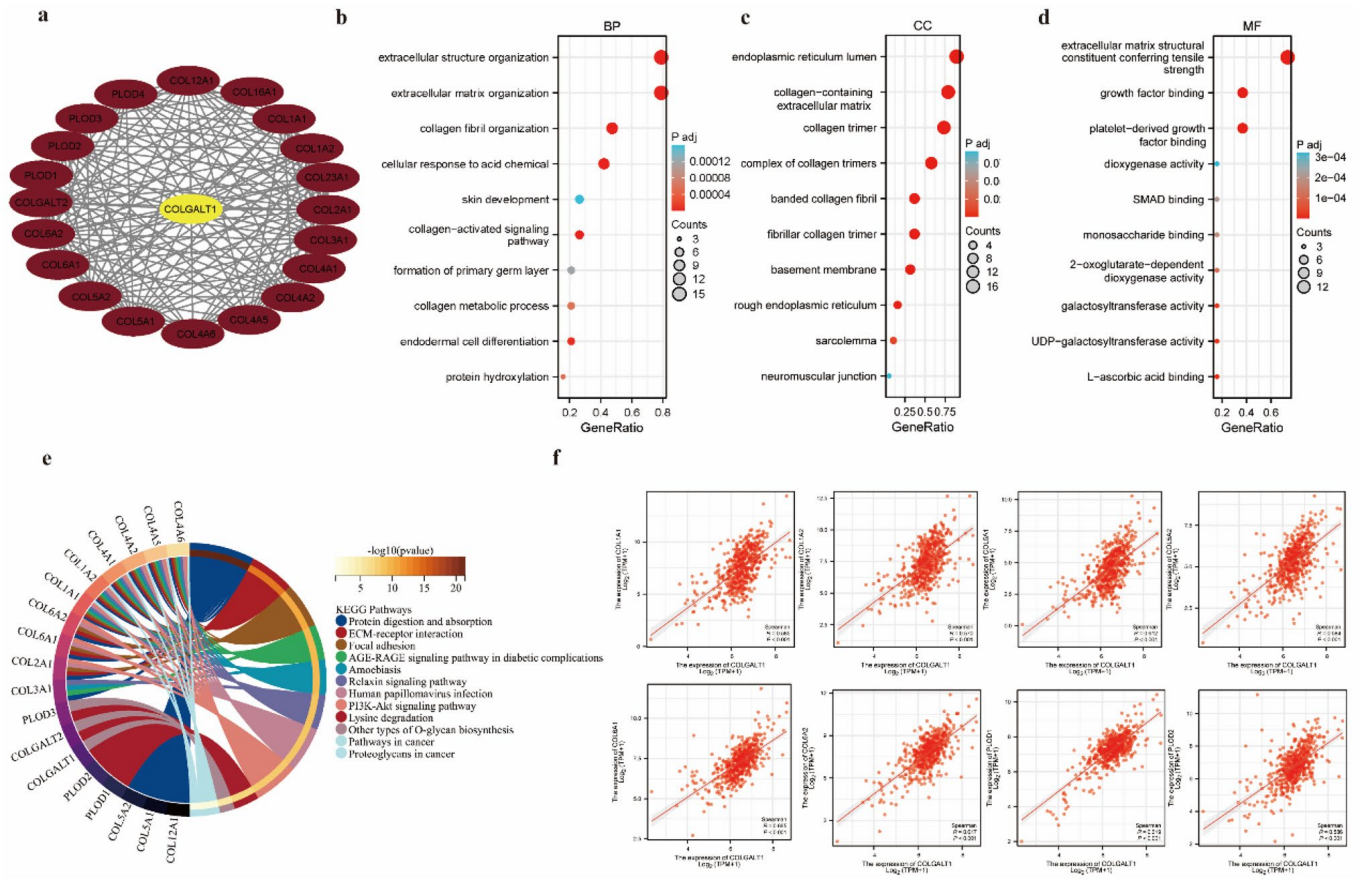
PPI network and functional enrichment analysis (**a**) COLGALT1 and its co-expressed gene PPI network. (**b**) BP (**c**) CC (**d**) MF (**e**) KEGG enrichment analysis of COLGALT1 and its 20 co-expressed genes. (**f**) Correlation analysis of COLGALT1 expression with co-expressed genes in ccRCC. BP Biological Process, CC Celluar Components, MF Molecular Functtion, KEGG Kyoto Encylopedia of Genes and Genomes

A PPI network incorporating COLGALT1 and its 20 co-expressed genes from the STRING database was created using “Cytoscape” (Fig. 4A). To explore the potential roles of COLGALT1 in ccRCC, GO and KEGG pathway enrichment analyses were performed on its top 20 associated genes (Fig. 4B, C). Correlation analysis revealed a positive relationship between COLGALT1 and its co-expressed genes (Fig. 4D). Based on the GO analysis, COLGALT1 was found to be linked to several biological processes. In terms of BP and MF, COLGALT1 was associated with external side of plasma membrane, and in CC, it was linked to endoplasmic reticulum lumen (Figs. 4B). The KEGG analysis revealed that COLGALT1 is involved in protein digestion and absorption, ECM-receptor interaction, focal adhesion and AGE-RAGE signaling pathway in diabetic complications (Fig. 4C). Functional analyses demonstrate that COLGALT1 participates in ECM organization, substantially contributing to ccRCC tumorigenesis and disease advancement.

### Association between COLGALT1 expression and immune infiltration in CcRCC

**Fig. 5:**
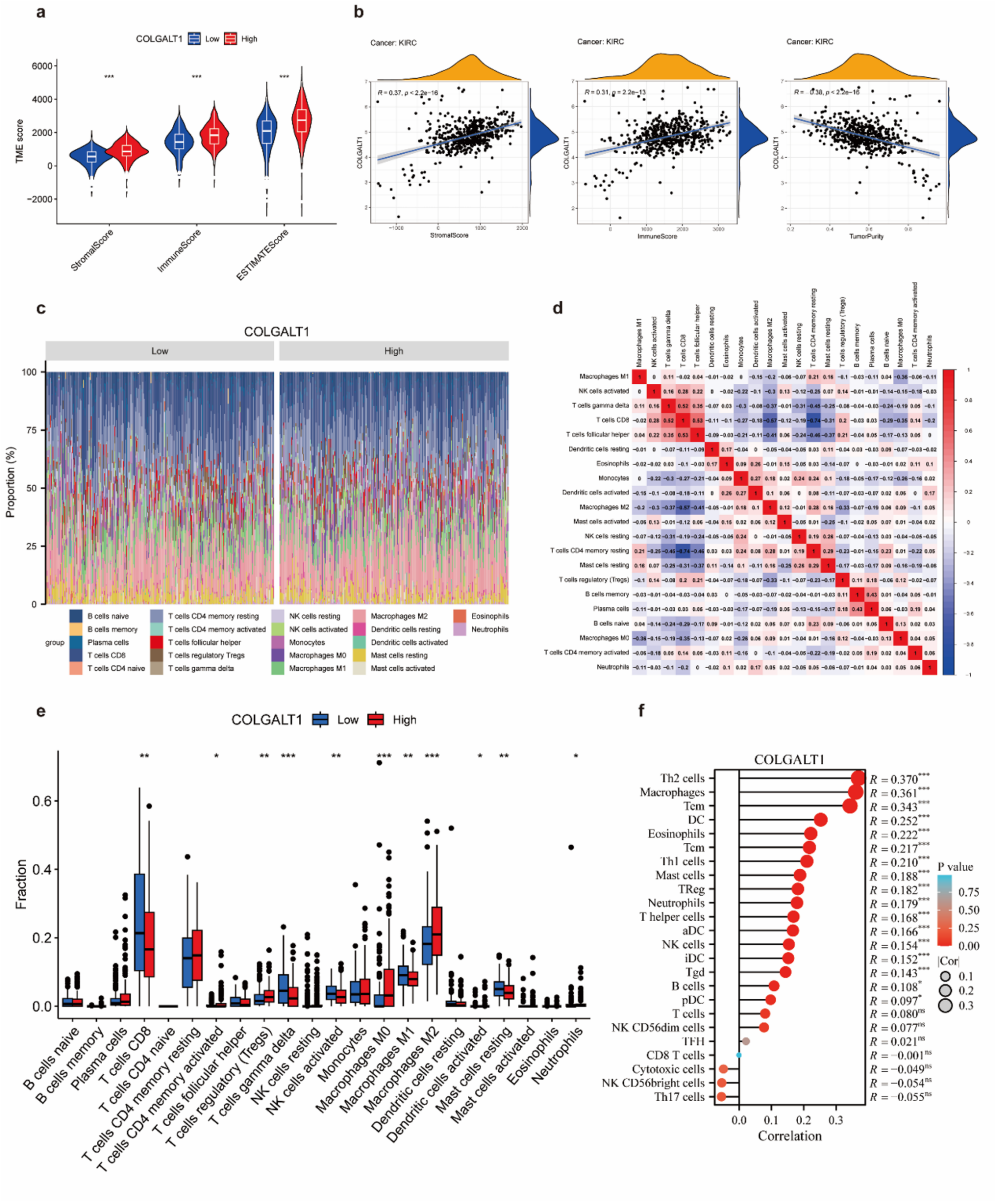
Association between COLGALT1 expression and immune cell infiltration in ccRCC (**a**) Stromal score, immune score, ESTIMATE score, and tumor purity were calculated using the ESTIMATE algorithm. (**b**) Analysis revealed that COLGALT1 expression in ccRCC was positively associated with stromal scores, as well as immune scores, and tumor purity. **c–e** The CIBERSORT algorithm was used to quantify 22 immune cell subsets in ccRCC samples. (**c**) A bar chart presents the relative proportion of immune cells in each sample, with different immune subsets distinguished by color. (**d**) Correlations between 21 immune cell types are shown, with statistical significance annotated. (**e**) Aviolin plot displays the distribution of each immune cell fraction across samples. (**f**) Single-sample Gene Set Enrichment Analysis (ssGSEA) of TCGA ccRCC samples demonstrated that COLGALT1 expression was related to the infiltration levels of multiple immune cell subtypes. (*P < 0.05, **P < 0.01, ***P < 0.001)

We explored the relationship between COLGALT1 expression and immune cell infiltration in ccRCC patients by employing multiple immune analysis algorithms, including ESTIMATE, CIBERSORT, and ssGSEA. Based on the average expression level of COLGALT1, 503 ccRCC transcriptome samples from TCGA were categorized into high and low expression groups.Results from the ESTIMATE algorithm indicated that patients with higher COLGALT1 expression had significantly elevated immune scores, stromal scores, and overall ESTIMATE scores (all *P* < 0.001) compared to those with lower expression (Fig. 5A). Additionally, COLGALT1 expression showed a positive correlation with stromal scores (*r* = 0.37, *P* < 0.001) and immune scores (*r* = 0.31, *P* < 0.001), and a negative correlation with tumor purity (*r* = -0.38, *P* < 0.001) (Fig. 5B).

Using the CIBERSORT algorithm, we generated box and violin plots illustrating the proportions of 22 immune cell types across ccRCC samples (Figs. 5C-E), further supporting the association between COLGALT1 expression and immune infiltration. Moreover, ssGSEA analysis revealed that most immune cell infiltration levels were positively correlated with COLGALT1 expression (Fig. 5F), especially Th2 cells, and macrophages with correlation coefficients exceeding 0.3.

To further confirm the link between COLGALT1 and tumor-infiltrating immune cells (TILs), we examined the relationship between COLGALT1 expression and various immune cell markers—including CD8+/CD4 + T cells, NK cells, B cells, monocytes, dendritic cells (DCs), tumor-associated macrophages (TAMs), M1/M2 macrophages, neutrophils, and exhausted T cells—using TIMER and TCGA data. Most TIL marker genes were significantly associated with COLGALT1 expression in ccRCC (Table 1). Notably, COLGALT1 expression was positively associated with key chemokines related to TAMs (IL10, CCL2, CD68) and Tregs (CCR8, FOXP3, TGFB1), and M2 macrophage markers (CD163, VSIG4, MS4A4A) (*P* < 0.001). These findings suggest that COLGALT1 may contribute to immune suppression in ccRCC by promoting M2 macrophage polarization.

**Table 1:** Correlation analysis between COLGALT1 and related genes and markers of immune cells in TIMER database

Description	Gene markers	KIRC Cor	*P*
CD8 T cell+	CD8A	0.131	**
	CD8B	0.101	*
	TBX21	0.257	***
	IFNG	0.092	*
	CXCL9	0.179	***
	CXCL10	0.095	*
T cell(general)	CD3D	0.142	***
	CD3E	0.197	***
	CD3G	0.226	***
	CD2	0.163	***
B cell	CD19	0.231	***
	CD79A	0.187	***
	BLK	0.228	***
Monocyte	CD86	0.383	***
	CD115(CSF1R)	0.564	***
TAM	CCL2	0.12	**
	CD68	0.336	***
	IL10	0.352	***
	CSF2	0.162	***
M1 Macrophage	INOS(NOS2)	0.334	***
	IRF5	0.242	***
	COX2(PTGS2)	0.276	***
M2 Macrophage	CD163	0.512	***
	VSIG4	0.564	***
	MS4A4A	0.456	***
Neutrophils	CD66b(CEACAM8)	-0.025	0.563
	CD11b(ITGAM)	0.509	***
	CCR7	0.334	***
Natural killer cell	KIR2DL1	0.141	***
	KIR2DL3	0.099	*
	KIR2DL4	0.137	***
	KIR3DL1	0.115	**
	KIR3DL2	0.107	**
	KIR3DL3	0.0467	0.276
	KIR2DL4	0.137	***
Dendritic cell	HLA-DPB1	0.246	***
	HLA-DQB1	0.196	***
	HLA-DRA	0.277	***
	HLA-DPA1	0.234	***
	CD1C	0.189	***
	NRP1	0.476	***
	ITGAX	0.287	***
Th1	TBX21	0.257	***
	STAT4	0.247	***
	STAT1	0.330	***
	IFN-g (IFNG)	0.092	*
	TNF-a (TNF)	0.209	***
Th2	GATA3	0.053	0.219
	STAT6	0.454	***
	STAT5A	0.454	***
	IL13	0.045	0.299
Tfh	BCL6	0.542	***
	IL21	0.134	**
Th17	STAT3	0.574	***
	IL17A	0.005	0.916
Treg	FOXP3	0.311	***
	CCR8	0.267	***
	STAT5B	0.387	***
	TGFB1	0.63	***
T cell exhaustion	PDCD1	0.14	***
	CD274	0.055	0.201
	CTLA4	0.175	***
	LAG3	0.176	***
	HAVCR2	0.085	*
	GZMB	0.184	***

### Single-cell landscape of COLGALT1 in CcRCC immunity

**Fig. 6:**
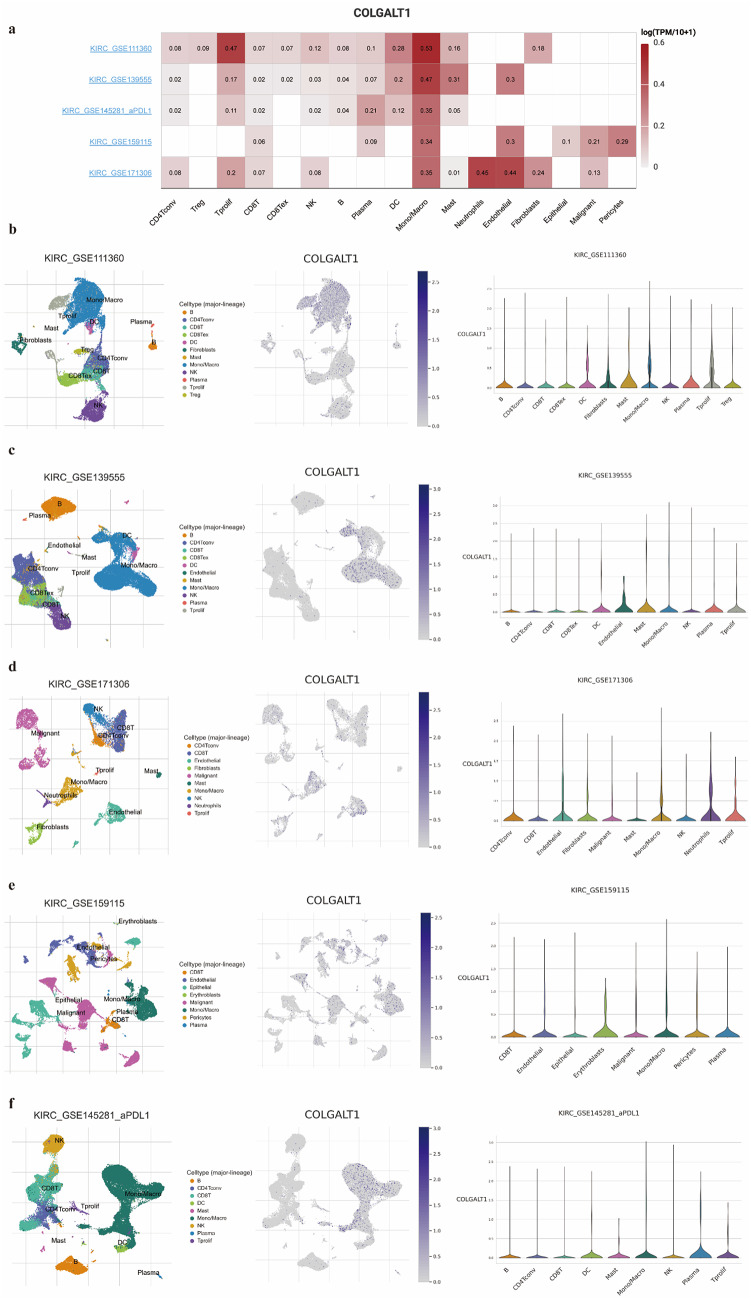
Single-cell analysis of the COLGALT1 and TME in ccRCC **a–f** Violin plots and UMAP plots of COLGALT1 and immune cell infiltration in (**a**) GSE111360 (**b**) GSE139555 (**c**) GSE145281 (**d**) GSE159115 (**e**) GSE17306

Single-cell transcriptomic profiling across five ccRCC cohorts (GSE111360, GSE139555, GSE159115, GSE171360, GSE145281) revealed COLGALT1’s cell-type-specific expression dynamics (Fig. 6A-F). While baseline tumors (GSE111360/139555/159115/171360) demonstrated COLGALT1 enrichment in NK cells, monocytes and macrophages, metastatic PD-L1-blockade-treated specimens (GSE145281) exhibited amplified COLGALT1 transcriptional activity in CD8 + T cells and therapy-refractory proliferating T cells (Tprolif), alongside activated NK clusters. Notably, sustained COLGALT1 expression in exhausted CD8 + T cell subsets across both untreated and immunotherapeutic contexts suggests its role in perpetuating T cell dysfunction—a hallmark of immune evasion. This COLGALT1-enriched exhausted T cell phenotype may foster an immunosuppressive niche, thereby counteracting PD-L1 blockade efficacy and promoting tumor progression.

### COLGALT1-driven immune regulatory networks

**Fig. 7:**
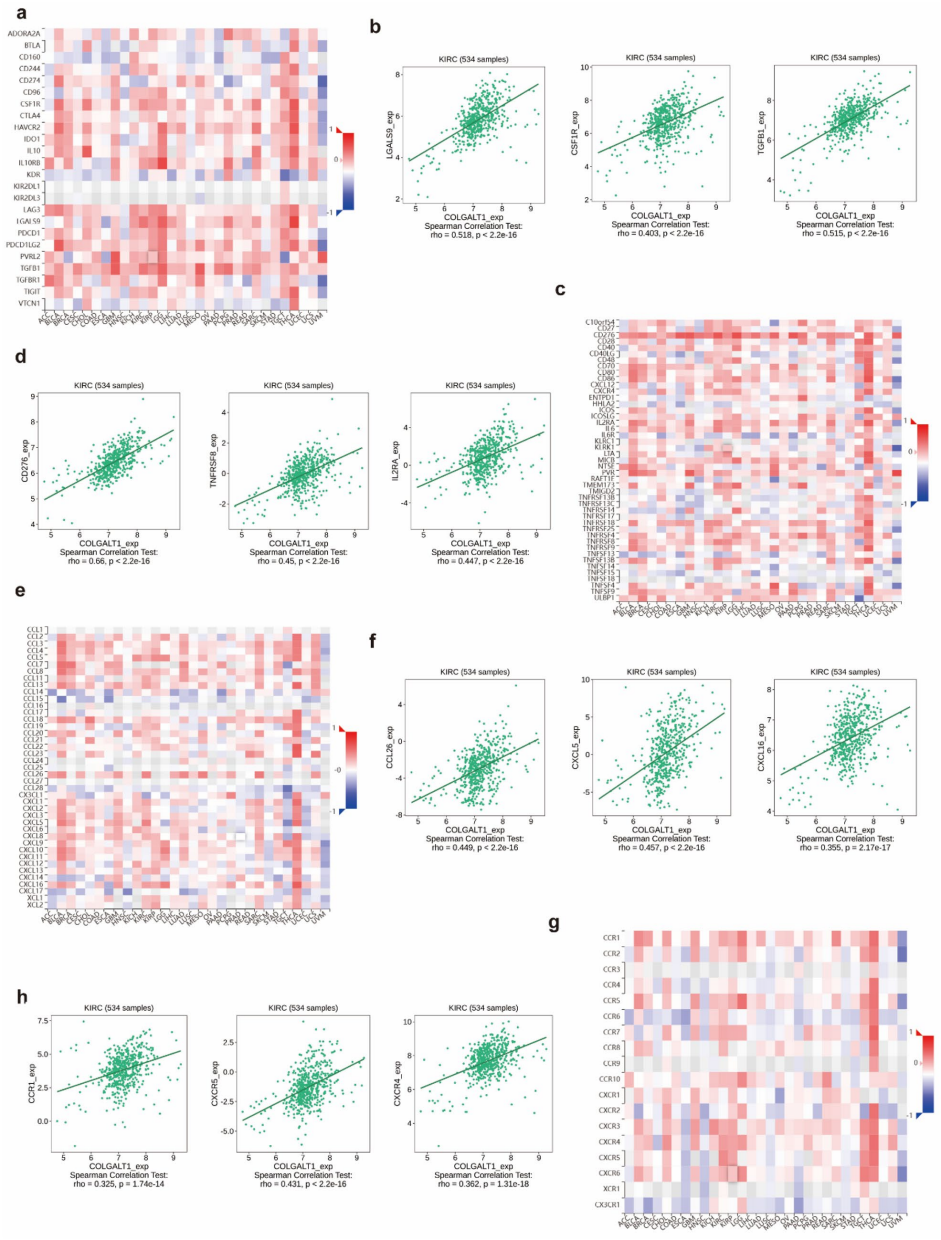
Association of COLGALT1 expression with immunoregulatory molecules and chemokine signaling components based on TISIDB analysis (**a**,** b**) COLGALT1 expression exhibited a positive correlation with the majority of immune inhibitory molecules. (**c**,** d**) A similar positive relationship was observed between COLGALT1 and various immune stimulatory factors. (**e**,** f**) COLGALT1 levels were also found to be positively linked with most chemokines. (**g**,** h**) Expression of COLGALT1 showed a strong positive association with numerous chemokine receptors

TISIDB-based systems immunology analysis revealed COLGALT1’s extensive coordination with immune modulators across four functional axes. Our findings indicated that COLGALT1 expression was positively correlated with most immunoinhibitors (Fig. 7A). Specifically, in ccRCC, the top three correlations were with LGALS9 (rho = 0.518), CSF1R (rho = 0.403), and TGFB1 (rho = 0.515) (Fig. 7B). Similarly, COLGALT1 expression showed a positive correlation with most immunostimulators (Fig. 7C), with the top three in ccRCC being CD276 (rho = 0.66), TNFRSF8 (rho = 0.45), and IL2RA (rho = 0.447) (Fig. 7D). COLGALT1 expression was also positively correlated with most chemokines (Fig. 7E), particularly CCL26 (rho = 0.449), CXCL5 (rho = 0.457), and CXCL16 (rho = 0.355) (Fig. 7F). Lastly, COLGALT1 expression showed a positive correlation with most chemokine receptors (Fig. 7G), with the top three in ccRCC being CCR1 (rho = 0.325), CXCR5 (rho = 0.431), and CXCR4 (rho = 0.362) (Fig. 7H).

### COLGALT1-centric CeRNA network construction and prognostic significance

To identify key miRNAs and lncRNAs associated with COLGALT1 in the TCGA–CCRCC dataset, we utilized the starBase v2.0 platform. The screening strategy included three main criteria: (1) selected miRNAs were negatively correlated with COLGALT1 expression; (2) selected lncRNAs showed a positive correlation with COLGALT1; and (3) candidate miRNAs were inversely correlated with the identified lncRNAs. Using starBase v2.0, which facilitates the analysis of mRNA, lncRNA, and miRNA interaction networks, we constructed a potential lncRNA/miRNA/COLGALT1 regulatory axis in CCRCC.

**Fig. 8:**
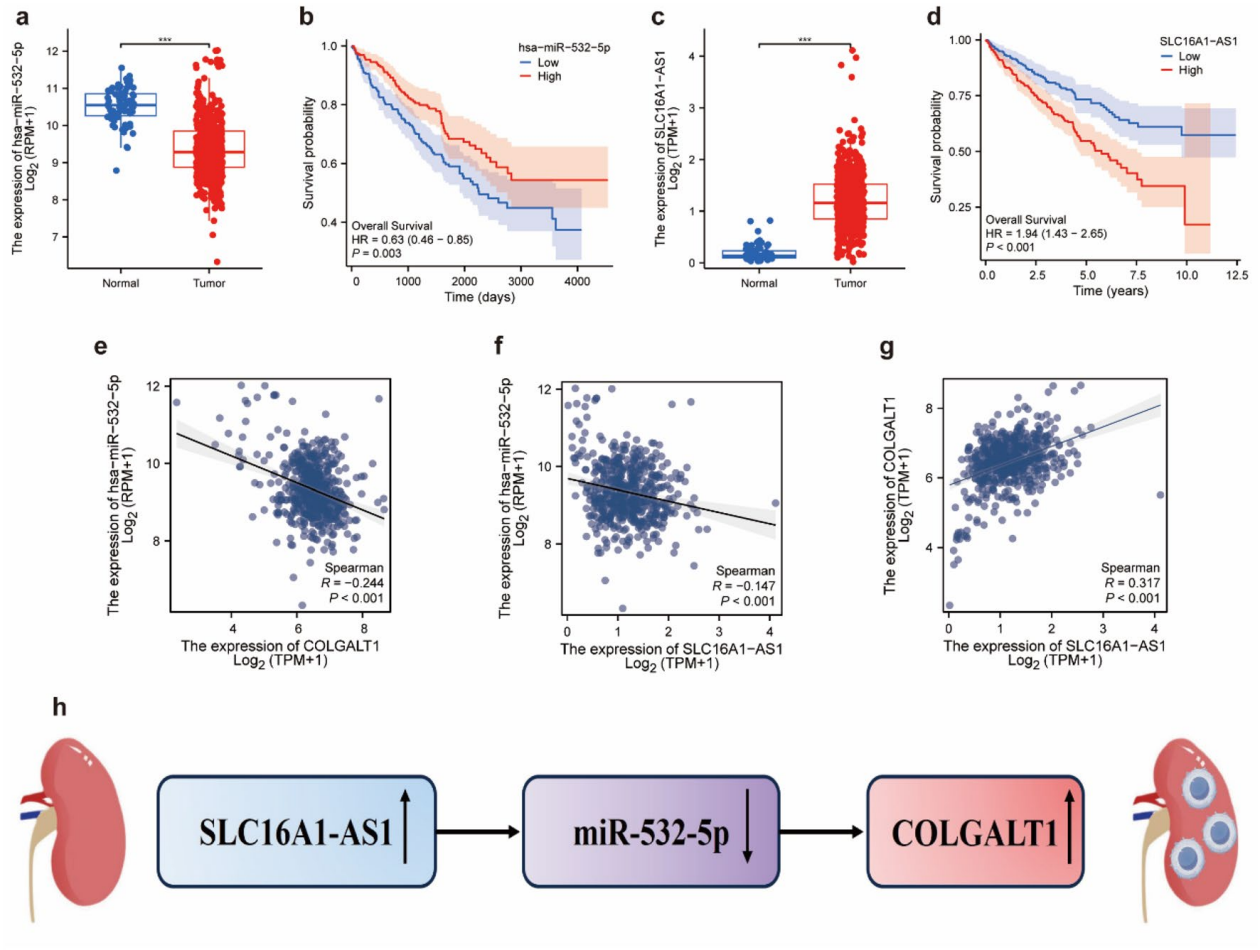
Construction and analysis of the potential SLC16A1-AS1/hsa-miR-532-5p/COLGALT1 ceRNA regulatory axis in the TCGA dataset (**a**) Expression analysis showed that hsa-miR-532-5p was significantly downregulated in ccRCC tissues compared to normal counterparts. (**b**) Kaplan–Meier survival analysis indicated a significant association between hsa-miR-532-5p expression and overall survival in ccRCC patients. (**c**) SLC16A1-AS1 was found to be upregulated in ccRCC samples relative to normal tissues. (**d**) High expression of SLC16A1-AS1 was correlated with poorer overall survival outcomes in ccRCC. (**e**) A negative correlation was observed between hsa-miR-532-5p and COLGALT1 expression. (**f**) SLC16A1-AS1 expression was inversely correlated with hsa-miR-532-5p levels. (**g**) A positive correlation was identified between SLC16A1-AS1 and COLGALT1 expression in ccRCC. (**h**) Predicted ceRNA network illustrating the regulatory interactions among SLC16A1-AS1, hsa-miR-532-5p, and COLGALT1 in the TCGA cohort

Subsequently, we validated our findings through further analysis. We observed that hsa-mir-502 expression was significantly lower in tumor tissues compared to normal tissues (*p* < 0.001), and higher levels of hsa-mir-502 were associated with improved patient survival (*p* = 0.003) (Fig. 8A, and B). Additionally, SLC16A1-AS1 was found to be markedly upregulated in tumor samples (*p* < 0.001), and patients with lower SLC16A1-AS1 expression had a more favorable prognosis than those with higher expression (*p* < 0.001) (Fig. 8C, and D).

Pearson correlation analysis based on TCGA data revealed that hsa-mir-502 was negatively associated with COLGALT1 (*R* = − 0.244, *p* < 0.001), SLC16A1-AS1 was inversely correlated with hsa-mir-502 (*R* = − 0.147, *p* < 0.001), and SLC16A1-AS1 was positively correlated with COLGALT1 (*R* = 0.317, *p* < 0.001) (Fig. 8E-G). Collectively, these findings suggest the existence of a potential ceRNA regulatory network involving SLC16A1-AS1, hsa-mir-502-3p, and COLGALT1 in ccRCC (Fig. 8H).

### Drug sensitivity

**Fig. 9:**
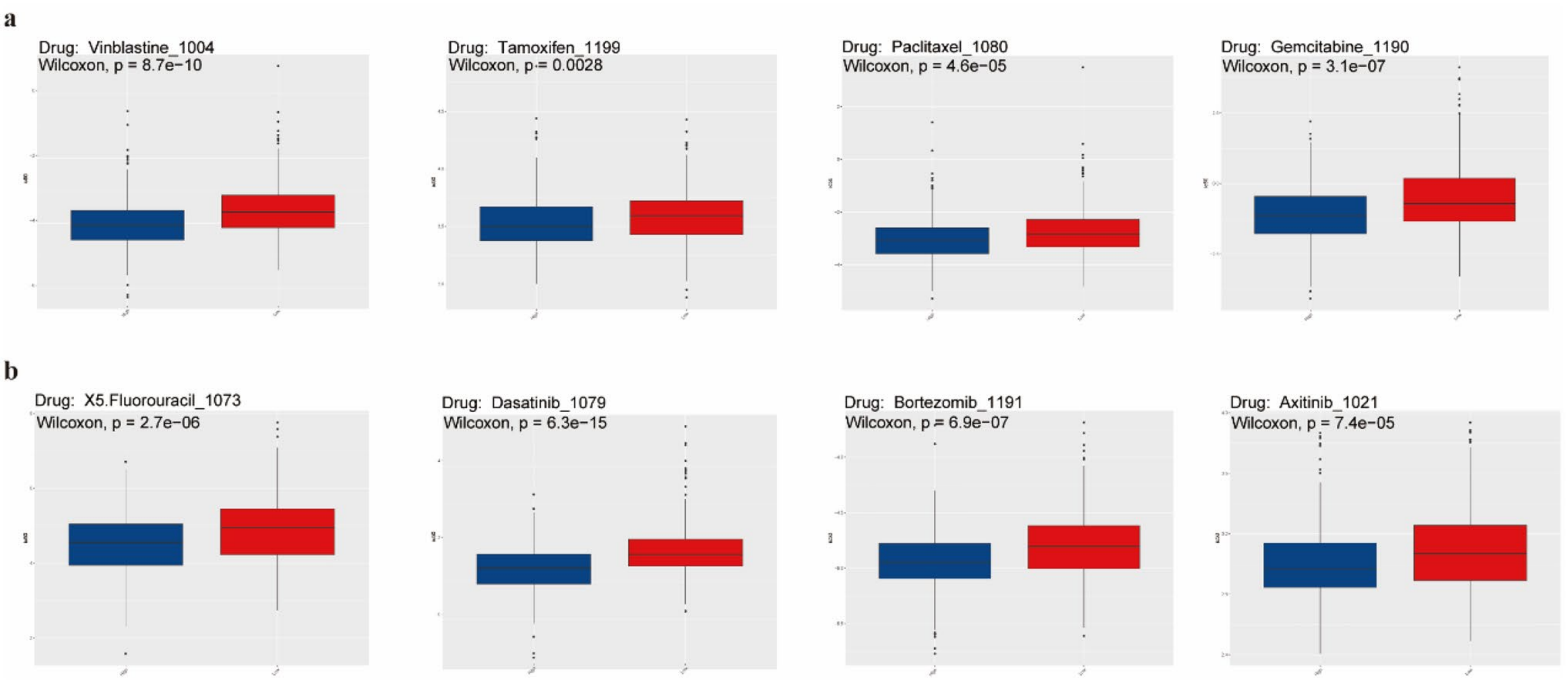
Comparison of immunotherapy and chemotherapy responses in patients with high vs. low COLGALT1 expression (**a**) Boxplots display the estimated drug sensitivity to Vinblastine, Tamoxifen, Paclitaxel, and Gemcitabine among patient groups stratified by COLGALT1 expression levels. (**b**) Drug response differences to Fluorouracil, Dasatinib, Bortezomib, and Axitinib are illustrated in boxplots comparing high and low COLGALT1 expression groups

To assess the predictive value of COLGALT1 in chemotherapy and targeted therapy outcomes, IC50 values for eight drugs were analyzed. Patients with elevated COLGALT1 expression demonstrated markedly higher IC50 levels for Vinblastine, Tamoxifen, Paclitaxel, Gemcitabine, Fluorouracil, Dasatinib, Bortezomib, and Axitinib compared to those with low COLGALT1, indicating increased drug resistance in the high-expression group (*P* < 0.05). (Fig. 9A, and B).

## Discussion

ccRCC represents the most prevalent and aggressive form of kidney cancer [25], frequently diagnosed at advanced stages due to its asymptomatic progression [26, 27], contributing to suboptimal clinical outcomes despite advancements in targeted and immune therapies [28]. The molecular heterogeneity of ccRCC underscores the critical need for biomarkers capable of guiding precision therapeutics [29]. In this context, biomarkers that not only predict prognosis but also reflect tumor–immune–stromal interactions are of particular clinical relevance.

COLGALT1, a collagen-modifying enzyme pivotal in ECM remodeling [9], has emerged as a key mediator of tumor-stroma crosstalk across malignancies [30]. Beyond its structural role, COLGALT1-mediated collagen glycosylation may influence mechanotransduction, metabolic signaling, and immune regulation within the tumor microenvironment. Our study provides comprehensive and integrative evidence linking COLGALT1 to tumor progression, immune remodeling, and adverse clinical outcomes in ccRCC.

By analyzing TCGA, GEO, and various online platforms, we observed that COLGALT1 expression was markedly elevated in ccRCC tissues compared to adjacent normal kidney tissues. These findings were further validated through both immunohistochemistry assay and qRT-PCR analysis. Additionally, higher COLGALT1 levels were significantly associated with more advanced tumor stages, higher pathological grades, and the presence of distant metastases, suggesting a potential oncogenic role for COLGALT1 in ccRCC, where its overexpression correlates with more aggressive clinical features. These results position COLGALT1 as a candidate feature for multivariable risk stratification rather than merely a standalone molecular marker.

To assess the clinical relevance of COLGALT1, we examined its impact on patient survival. Kaplan-Meier survival analysis indicated that elevated COLGALT1 expression was significantly linked to poorer OS, DSS, and PFI, which supports its potential as a prognostic biomarker in ccRCC. Furthermore, Time-dependent ROC curve and multivariate Cox regression analysis identified COLGALT1 as an independent prognostic factor, regardless of other clinical characteristics. From a predictive modeling perspective, these findings suggest that COLGALT1 could be incorporated into supervised learning frameworks together with clinicopathological and immune-related variables to improve prognostic accuracy and individualized risk prediction.

Although ccRCC is known for its high degree of immune cell infiltration [31, 32], the connection between COLGALT1 and the immune landscape of ccRCC had not been previously examined. Our study addressed this gap by evaluating the relationship between COLGALT1 expression and the tumor immune microenvironment. Immune infiltration analysis revealed positive correlations between COLGALT1 and several TIL populations, including NK cells, TAMs, Tregs, and macrophages. Moreover, COLGALT1 expression was associated with various immune-related molecules, such as chemokines, receptors, immunostimulatory, and immunosuppressive factors. Cross-validation across multiple immune deconvolution algorithms and single-cell datasets revealed consistent enrichment of immunosuppressive immune subsets, supporting the robustness of these associations. We also explored its relationship with marker genes of TILs, further suggesting that COLGALT1 is more closely linked to immune remodeling and immune dysfunction rather than effective anti-tumor immunity within the TME.

Macrophages, key components of the immune landscape, can polarize into either pro-inflammatory M1 or immunosuppressive M2 phenotypes [33]. M1 macrophages, typically activated by bacterial components or Th1 cytokines, are associated with anti-tumor effects and favorable outcomes [34, 35]. In contrast, M2 macrophages, stimulated by Th2 cytokines, contribute to tumor growth, angiogenesis, and immune suppression [36, 37]. In our study, COLGALT1 expression showed a positive correlation with M2 macrophage markers (VSIG4, MS4A4A) but not with M1 markers (NOS2, COX2), implying that COLGALT1 may play a role in skewing macrophage polarization toward a tumor-promoting M2 phenotype in ccRCC. This macrophage polarization bias may be mechanistically linked to ECM-driven signaling pathways and immunometabolic reprogramming within the tumor microenvironment.

The ceRNA network has been recognized as a critical factor in cancer initiation and progression [38]. These networks function through non-coding RNAs (ncRNAs) that modulate gene expression by competing for shared microRNA binding sites [39, 40]. In this study, we used bioinformatics analysis to construct a ceRNA network related to COLGALT1 deletion, which may affect ccRCC cell proliferation. Key ncRNAs including SLC16A1-AS1 and miR-532-5p were identified, both previously linked to cancer, including ccRCC. Notably, reduced miR-532-5p expression is associated with increased cell migration, invasion, and proliferation [41]. These regulatory interactions provide an additional layer of post-transcriptional control that may contribute to COLGALT1 dysregulation and tumor progression.

Importantly, ccRCC is a metabolically driven malignancy characterized by mitochondrial dysfunction, altered oxidative metabolism, and dysregulated mTOR signaling. Although not directly examined in this study, COLGALT1-mediated ECM remodeling may indirectly influence mitochondrial homeostasis and mitochondrial DNA stability through mechanotransduction and stromal–immune signaling [42]. Furthermore, crosstalk between ECM–focal adhesion signaling and mTOR activation may promote immunosuppressive macrophage polarization and T cell dysfunction, providing a plausible mechanistic link between COLGALT1 overexpression, immune remodeling, and poor clinical outcomes.

Nevertheless, our study has several limitations that should be acknowledged. Firstly, the majority of analyses were based on publicly available datasets, which, although comprehensive, may introduce biases related to data heterogeneity and patient selection. Validation in larger, independent clinical cohorts is therefore warranted to strengthen the robustness of our findings. Secondly, while our in vitro experiments provided preliminary functional evidence supporting the role of COLGALT1, additional in vivo studies are necessary to confirm its impact on tumor growth, immune modulation, and therapeutic response under physiological conditions. Thirdly, the precise molecular mechanisms through which COLGALT1 influences immune infiltration, mitochondrial function, and mTOR-related signaling pathways remain largely unexplored. Future mechanistic studies integrating genetic manipulation, metabolic profiling, and immunological assays will be essential to delineate these pathways. Despite these limitations, our integrated bioinformatics and experimental approach provide the first comprehensive evidence linking COLGALT1 to prognosis, immune regulation, and therapeutic response in ccRCC, thereby laying the groundwork for future translational investigations.

## Conclusion

In summary, our findings highlight COLGALT1 as a promising prognostic biomarker and potential therapeutic target in ccRCC. It appears to influence the immune microenvironment and may modulate responses to immunotherapy. Beyond its prognostic value, COLGALT1 may serve as a biologically informative feature for integrative predictive modeling and therapeutic stratification. Future research should focus on clinical validation and investigating COLGALT1-targeted therapies, possibly in combination with current immunotherapeutic strategies, to enhance treatment outcomes in ccRCC.

## Supplementary Information

Below is the link to the electronic supplementary material.

Supplementary Material 1

Supplementary Material 2

Supplementary Material 3

Supplementary Material 4

Supplementary Material 5

## Data Availability

All data are obtained in the article.
