# Supplementary material for: Comprehensive analysis of COLGALT1 in tumor microenvironment regulation and prognosis of clear cell renal cell carcinoma
Source: Clin Exp Med. 2026 Feb 2;26(1):127. doi: 10.1007/s10238-026-02041-6 (PMC12872700; doi:10.1007/s10238-026-02041-6)
Supplement: Supplementary file 1 — Supplementary Material 1 [file 10238_2026_2041_MOESM1_ESM.docx]

Supplementary Table 1. Primer sequences for qRT-PCR

| Gene (Human) | Forward primer (5’~3’) | Reverse primer (5’~3’) |
| --- | --- | --- |
| COLGALT1 | GATGCTGCCTGTGGACGAGTTC | CTCACATAGCCATCGTCTCCTG |
| β-actin | GTCTCCTCTGACTTCAACAGCG | ACCACCCTGTTGCTGTAGCCAA |
