# Supplementary material for: Comprehensive analysis of COLGALT1 in tumor microenvironment regulation and prognosis of clear cell renal cell carcinoma
Source: Clin Exp Med. 2026 Feb 2;26(1):127. doi: 10.1007/s10238-026-02041-6 (PMC12872700; doi:10.1007/s10238-026-02041-6)
Supplement: Supplementary file 4 — Supplementary Material 4 [file 10238_2026_2041_MOESM4_ESM.docx]

Supplementary table 2. Association between COLGALT1 expression and clinicopathologic features in ccRCC samples from the TCGA.

| Characteristics | Low expression of COLGALT1 | High expression of COLGALT1 | P value |
| --- | --- | --- | --- |
| n | 270 | 271 |  |
| Pathologic T stage, n (%) |  |  | 0.052 |
| T1 | 142 (26.2%) | 137 (25.3%) |  |
| T2 | 42 (7.8%) | 29 (5.4%) |  |
| T3 | 84 (15.5%) | 96 (17.7%) |  |
| T4 | 2 (0.4%) | 9 (1.7%) |  |
| Pathologic N stage, n (%) |  |  | 0.061 |
| N0 | 119 (46.1%) | 123 (47.7%) |  |
| N1 | 4 (1.6%) | 12 (4.7%) |  |
| Pathologic M stage, n (%) |  |  | 0.061 |
| M0 | 223 (43.9%) | 206 (40.6%) |  |
| M1 | 32 (6.3%) | 47 (9.3%) |  |
| Pathologic stage, n (%) |  |  | 0.183 |
| Stage I | 139 (25.8%) | 134 (24.9%) |  |
| Stage II | 35 (6.5%) | 24 (4.5%) |  |
| Stage III | 62 (11.5%) | 61 (11.3%) |  |
| Stage IV | 34 (6.3%) | 49 (9.1%) |  |
| Histologic grade, n (%) |  |  | 0.031 |
| G1 | 4 (0.8%) | 10 (1.9%) |  |
| G2 | 125 (23.5%) | 111 (20.8%) |  |
| G3 | 107 (20.1%) | 100 (18.8%) |  |
| G4 | 28 (5.3%) | 48 (9%) |  |
| Age, n (%) |  |  | 0.366 |
| <= 60 | 129 (23.8%) | 140 (25.9%) |  |
| > 60 | 141 (26.1%) | 131 (24.2%) |  |
| Gender, n (%) |  |  | 0.335 |
| Female | 88 (16.3%) | 99 (18.3%) |  |
| Male | 182 (33.6%) | 172 (31.8%) |  |
| Race, n (%) |  |  | 0.354 |
| White | 238 (44.6%) | 231 (43.3%) |  |
| Asian&Black or African American | 29 (5.4%) | 36 (6.7%) |  |
| Laterality, n (%) |  |  | 0.487 |
| Left | 122 (22.6%) | 131 (24.3%) |  |
| Right | 147 (27.2%) | 140 (25.9%) |  |
| OS event, n (%) |  |  | 0.326 |
| Alive | 188 (34.8%) | 178 (32.9%) |  |
| Dead | 82 (15.2%) | 93 (17.2%) |  |
| DSS event, n (%) |  |  | 0.176 |
| No | 216 (40.8%) | 205 (38.7%) |  |
| Yes | 48 (9.1%) | 61 (11.5%) |  |
| PFI event, n (%) |  |  | 0.272 |
| No | 195 (36%) | 184 (34%) |  |
| Yes | 75 (13.9%) | 87 (16.1%) |  |
