# Supplementary material for: Comprehensive analysis of COLGALT1 in tumor microenvironment regulation and prognosis of clear cell renal cell carcinoma
Source: Clin Exp Med. 2026 Feb 2;26(1):127. doi: 10.1007/s10238-026-02041-6 (PMC12872700; doi:10.1007/s10238-026-02041-6)
Supplement: Supplementary file 5 — Supplementary Material 5 [file 10238_2026_2041_MOESM5_ESM.docx]

Supplemental Table 3 Univariate and Multivariate Cox Regression Analysis of Factors Associated with OS in ccRCC

|  | Total(N) | Univariate analysis | |  | Multivariate analysis | |
| --- | --- | --- | --- | --- | --- | --- |
|  |  | Hazard ratio (95% CI) | P |  | Hazard ratio (95% CI) | P |
| Age | 541 |  |  |  |  |  |
| <= 60 | 269 | Reference |  |  |  |  |
| > 60 | 272 | 1.745 (1.319 - 2.432) | **< 0.001** |  |  |  |
| Gender | 541 |  |  |  |  |  |
| Female | 187 | Reference |  |  |  |  |
| Male | 354 | 0.919 (0.679 - 1.257) | 0.613 |  |  |  |
| Pathologic stage | 538 |  |  |  |  |  |
| Stage I&Stage II | 332 | Reference |  |  | Reference |  |
| Stage III&Stage IV | 206 | 3.910 (2.852 - 5.360) | **< 0.001** |  | 2.706 (1.980 - 3.442) | **< 0.001** |
| Histologic grade | 533 |  |  |  |  |  |
| G1&G2 | 250 | Reference |  |  | Reference |  |
| G3&G4 | 283 | 2.563 (1.898 - 3.643) | **< 0.001** |  | 1.743 (1.224 - 2.509) | **0.002** |
| COLGALT1 | 541 |  |  |  |  |  |
| Low | 270 | Reference |  |  | Reference |  |
| High | 271 | 2.764 (1.914 – 3.486) | **< 0.001** |  | 1.898 (1.396 - 2.519) | **0.001** |
